# New Insights in (Inter)Cellular Mechanisms by Heart Failure with Preserved Ejection Fraction

**DOI:** 10.1007/s11897-014-0219-3

**Published:** 2014-09-05

**Authors:** Carsten Tschöpe, Sophie Van Linthout

**Affiliations:** 1 Department of Cardiology and Pneumology, Charité, University Medicine Berlin, Campus Benjamin Franklin, Hindenburgdamm 30, 12200 Berlin, Germany; 2 Berlin-Brandenburg Center for Regenerative Therapies, Charité, University Medicine Berlin, Campus Virchow Clinic, Berlin, Germany

**Keywords:** Endothelial dysfunction, Comorbidities, HFpEF, Cardiac endothelium, Nitric oxide, TGF-ß, Endothelial-to-mesenchymal transition, NOX, Inflammation, Cardiac fibrosis, Myofibroblast, Cardiomyocyte hypertrophy, Cardiomyocyte stiffness, Titin, Protein kinase A, Protein kinase G, cGMP, Sildenafil, Neurohumoral activation, Natriuretic peptides, BNP paradox, Neprilysin inhibitor, Mesenchymal stromal cells, Immunomodulation, Bidirectional cell interactions

## Abstract

Recently, a new paradigm for the development of heart failure with preserved ejection fraction (HFpEF) has been proposed, which identifies a systemic pro-inflammatory state induced by comorbidities as the origin of microvascular endothelial cell inflammation and subsequent concentric cardiac remodeling and dysfunction. This review further discusses the pivotal role of the inflamed endothelium in the pathogenesis of HFpEF-specific cardiac remodeling. The potential importance of reciprocal interactions of the endothelium with cardiac fibroblasts and cardiomyocytes and with the cardiac neurohumoral response in this cardiac remodeling process is outlined.

## Introduction

Epidemiological studies demonstrate that 50 % of all heart failure patients suffer from heart failure with preserved ejection fraction (HFpEF), which—in contrast to heart failure with reduced ejection fraction (HFREF)—cannot be adequately treated with current available therapeutical strategies [1]. Diabetes mellitus, obesity, hypertension, and COPD are the main comorbidities associated with HFpEF. [2] The high prevalence of HFpEF on the one hand and the rising prevalence of diabetes mellitus [3] and obesity [4] on the other hand, indicate the need for HFpEF-specific therapies. Further understanding of the underlying pathogenesis of HFpEF is required in view of finding novel treatment options. Diastolic stiffness underlying HFpEF is attributed to excessive myocardial collagen deposition and cardiomyocyte stiffness [5], of which the latter newly has been shown to be sufficient to induce HFpEF without any involvement of the extracellular matrix [6•]. Recently, a novel paradigm was postulated which identifies a systemic pro-inflammatory state induced by comorbidities as the origin of microvascular endothelial cell inflammation, which triggers HFpEF-specific, i.e., concentric, cardiac remodeling, and dysfunction [7••].

**Fig. 1:**
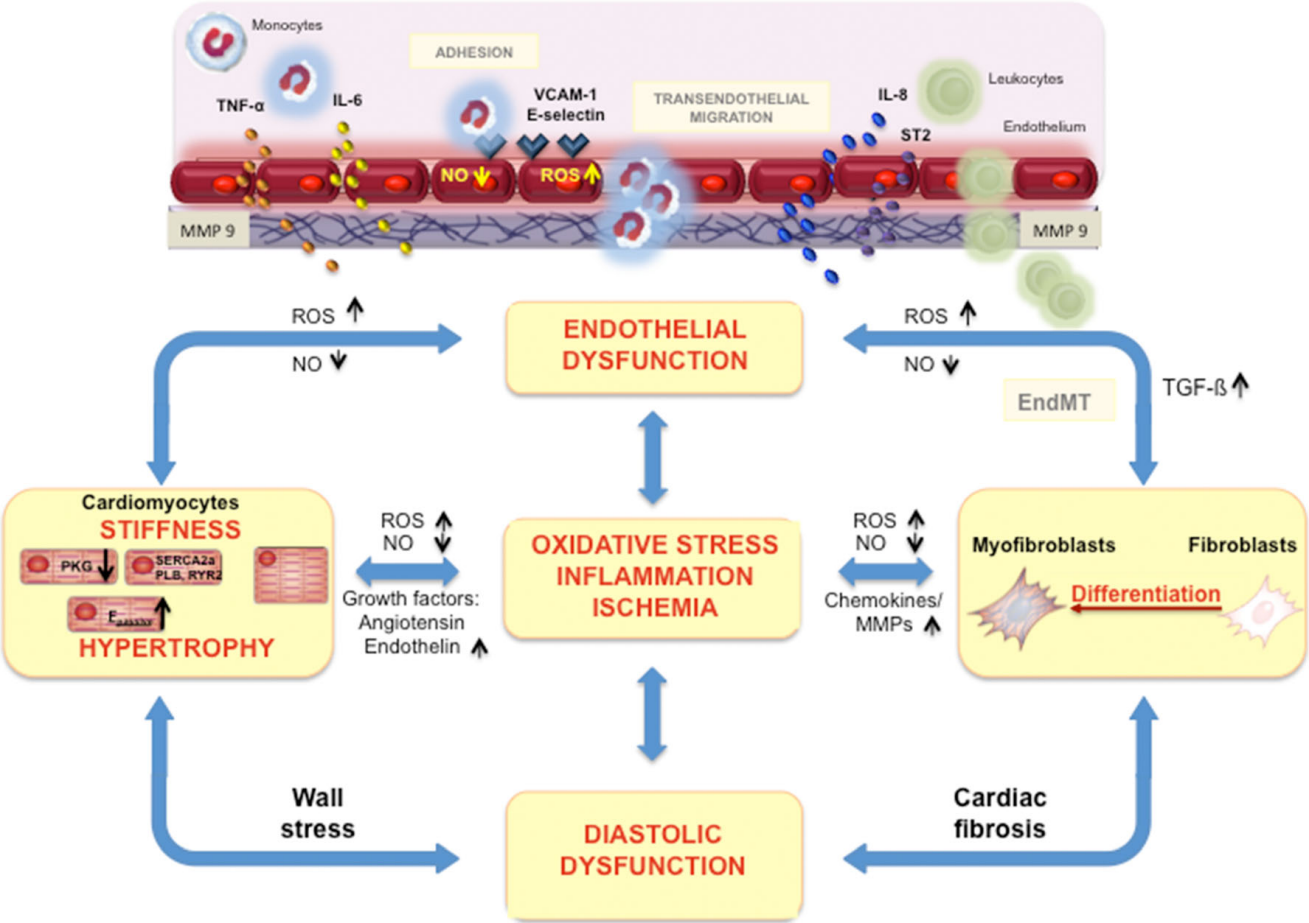
*Impact of endothelial dysfunction on the pathogenesis of HFpEF.* Comorbodities such as obesity, hypertension, diabetes mellitus, microalbuminuria, iron deficiency, and hypercoagulability induce a systemic inflammatory state leading to increased VCAM-1 expression, subsequent monocyte adhesion, and infiltration of inflammatory cells releasing pro-inflammatory factors including interleukin (IL)-6, IL-8, tumor necrosis factor (TNF)-α, and soluble ST2 (sST2), which promote endothelial dysfunction. The resulting endothelial dysfunction is associated with impaired NO bioavailibilty and ROS production, leading to the formation of peroxynitrite (ONOO^−^), all of which induce lowering of protein kinase G (PKG) activity and subsequently titin hypophosphorylation and an increased resting tension (*F*_passive_) of cardiomyocytes. Intracellular calcium dysregulation involving dysfunction of the sarcoplasmic reticular adenosine triphosphate (ATP)-driven pump (SERCA), phospholamban (PLB), and/or ryanodine receptors (RYR) 2 further participates to cardiomyocyte stiffness. Low PKG activity and, among others, the release of endothelin-1 by activated endothelial cells, contribute to cardiomyocyte hypertrophy. Furthermore, NO deprivation results in endothelial-to-mesenchymal transition (EndMT), a process by which endothelial cells transdifferentiate into (myo)fibroblasts. VCAM-1 and E-selectin expression on endothelial cells favors the adhesion of leukocytes, which by releasing transforming growth factor β (TGF-β) stimulate EndMT and the conversion of fibroblasts to myofibroblasts and hereby cardiac fibrosis. By the release of, among others, chemokines (monocyte chemotractant protein-1) and the induction of matrix metalloproteinases, (myo)fibroblasts stimulate the inflammatory process. Cardiac fibrosis, cardiomyocyte hypertrophy, and/or cardiomyocyte stiffness contribute to wall stress and diastolic dysfunction

This review gives a brief overview on how systemic inflammation induced by comorbidities influences endothelial cell behavior and signaling and how this affects the interaction of the endothelium with cardiomyocytes and cardiac fibroblasts, and the cardiac neurohumoral response, and subsequent cardiac remodeling. On the other hand, this review points out that those cells in turn influence the inflammation or directly the endothelium, aggravating the initial inflammatory process (Fig. 1). The impact of changes in substrate and subsequent energy metabolism on the pathogenesis of HFpEF is beyond the scope of this review.

### Endothelial Dysfunction and Heart Failure with Preserved Ejection Fraction

The endothelium, the physical barrier between the blood and vascular wall, plays a pivotal role in cardiovascular homeostasis by regulating vasomotor tone, vascular permeability, and cardiac function [8]. The release of nitric oxide (NO) is crucial for the normal function of the endothelium: NO mediates vasodilatation, inhibits platelet aggregation, and protects the integrity of the endothelial layer via its anti-inflammatory [9–12], anti-apoptotic [13], and pro-angiogenic [14] properties.

The HFpEF-associated comorbidities lead to endothelial dysfunction, a condition characterized by impaired endothelium-dependent vasodilatation and “endothelial activation,” referring to a state in which the endothelium loses its physiological properties and shifts towards a pro-inflammatory, pro-coagulatory, and vasoconstrictor state [15]. Particularly hyperglycemia, activation of the renin-angiotensin system, and tumor necrosis factor alpha (TNF-α) underlie decreased NO bioavailability, the main characteristic of endothelial dysfunction, involving (1) deprivation of the substrate L-arginine via impaired recycling of L-citrulline [16]; (2) increased expression/activity of the natural competitor of endothelial NO synthase (eNOS), arginase [17]; (3) reduced expression of eNOS [12, 18]; (4) a decreased ratio of the eNOS dimer to monomer [19]; (5) deprivation of the co-factor tetrabiopterin [20]; and/or (6) eNOS uncoupling, a phenomena by which eNOS produces superoxide (O_2_•) rather than NO [19, 21]. Hyperglycemia- or dyslipidemia-induced nicotinamide adenine dinucleotide phosphate (NAD(P)H) oxidase (NOX) [22] leads to O_2_• production and subsequent formation of peroxynitrite (ONOO–), which enhances eNOS uncoupling via reducing the affinity of eNOS for tetrabiopterin and via limiting the de novo synthesis of tetrabiopterin [23].

The cardiac endothelium comprises the endothelial cells of the coronary microvasculature, of the endocardium, and of the intramyocardial capillaries. Cardiomyocytes lie within the coronary microvascular network maximum 3 μm from endothelial cells. This specific anatomical arrangement allows not only adequate blood supply but also facilitates the bidirectional communication among those cells [24–26]. The relevance of the cardiac endothelium in cardiac function follows from pioneer work from Brutsaert et al. [27] who demonstrated in vitro that the cardiac endocardial surface modulates the performance of cardiac muscle. Via coronary infusion of substance P leading to acute modulation of left ventricular (LV) function, Paulus et al. [28] provided later evidence that the cardiac endothelium is important for cardiac contractile function in humans potentially via the release of paracrine factors including NO, endothelin-1, natriuretic peptides, cytokines, and others. Both studies accentuate the significance of the cardiac endothelium for (acute) cardiomyocyte function. How the cardiac endothelium and consequently endothelial dysfunction or deterioration affects cardiomyocytes as well as cardiac fibroblasts and cardiac neurohumoral activation will further be addressed in the following paragraphs. The impact of the cardiac endothelium on those different cardiac cells along with complex (bidirectional) interactions accentuates the relevance of endothelial dysfunction [29, 30] in the pathogenesis of HFpEF.

### Impact of Endothelial Dysfunction on Cardiac Fibroblasts

#### Cardiac Inflammation Triggers Cardiac Fibroblasts

Endothelial dysfunction/activation may contribute to cardiac fibrosis by different means. Murdoch et al. [31] recently demonstrated the role of endothelial NOX2 in the induction of cardiac inflammation and fibrosis in angiotensin II (Ang II) mice. Endothelium NOX2 transgenic mice had more vascular cell adhesion molecule (VCAM)-1 positive blood vessels and inflammatory cells after Ang II treatment than wild-type mice. In vitro co-culture of endothelial cells overexpressing NOX2 with cardiac fibroblasts provided further evidence that the induction of endothelial oxidative stress on its own is not responsible for the induction of cardiac fibrosis rather the stimulated inflammatory process. Since NO is capable of reducing oxidative stress on the one hand and decreasing the expression of monocyte chemoattractant protein-1 [9], VCAM-1 [10], and subsequent adhesion of immune cells [11] on the other hand, impaired NO bioavailability facilitates the engraftment of immune cells. Subsequently, engrafted immune cells may trigger cardiac fibrosis via inducing the differentiation of fibroblasts into myofibroblasts, which is reviewed in detail elsewhere [32••]. The importance of inflammation in the induction of cardiac fibrosis and heart failure, and particularly the role of the spleen as monocyte reservoir, has recently been broadly demonstrated [33–35]. In brief, we demonstrated that upon co-culture with fibroblasts, splenocytes isolated from mice with inflammatory cardiomyopathy induce a higher collagen production in fibroblasts compared to splenocytes from control mice [34]. Ismahil et al. [33] showed that myocardial infarction is associated with cardiac inflammation and concomitant depletion of splenic monocytes. Furthermore, splenectomy experiments and adoptive transfer of splenocytes from mice with heart failure in naive recipients illustrated that splenocytes retain memory upon adoptive transfer and subsequently promote immune-mediated fibrosis in the failing heart. Later, van der Laan et al. [35] confirmed this unique spatiotemporal pattern of monocyte accumulation in the myocardium following myocardial infarction, which coincides with a marked depletion of monocytes from the spleen, in humans via analysis of human post-mortem specimens of myocardium, spleen, and bone marrow. Similar to splenocytes from myocarditis mice [34], we demonstrated that co-culture of splenocytes from Ang II-induced HFpEF mice induced a 46 % higher collagen content in fibroblasts compared to splenocytes from control mice. This suggests that also in the pathogenesis of HFpEF, the cardiosplenic axis may be relevant. With respect to HFpEF, Westermann et al. [36] provided clear evidence linking cardiac inflammation with TGF-β1-induced collagen synthesis and diastolic dysfunction in human subjects [37]. The role of TGF-ß as a pro-fibrotic factor and its importance in the induction of diastolic function follows from previous experiments whereby the use of an anti-TGF-ß-neutralizing antibody prevented myocardial fibrosis and diastolic dysfunction in pressure-overloaded rats [38].

Besides immune cells, also fibrocytes, circulating monocyte-derived cells with tissue remodeling properties of fibroblasts [39], which are induced in cardiovascular disorders [39, 40], can adhere to the activated endothelium, engraft in the heart, and contribute to cardiac fibrosis.

#### Impaired Nitric Oxide Bioavailability Triggers Cardiac Fibroblasts

Secondly, endothelial dysfunction contributes to cardiac fibrosis via the reduced bioavailability of NO, known to exert direct anti-fibrotic effects involving the cyclic guanosine monophosphate (cGMP) pathway [41, 42]. Vettel et al. [42] recently demonstrated that the cGMP/cyclic adenosine monophosphate (cAMP)-hydrolyzing phosphodiesterase (PDE) 2, which is upregulated in human failing hearts [43], leads to a decrease in cAMP levels in cardiac fibroblasts and accelerates the conversion of fibroblasts to myofibroblasts. Exogenous activation of cGMP via atrial natriuretic peptide (ANP) or NO was able to bypass the PDE2-mediated degradation of cAMP and reversed the differentiation of fibroblasts into myofibroblasts. These data emphasize the benefit of cGMP-elevating agents in the treatment of HFpEF which, besides their well-recognized merit for cardiomyocytes, can also be beneficial for cardiac fibroblasts. PDE5A inhibition with sildenafil counteracted cardiac hypertrophy and adverse remodeling in Ang II mice, an effect which was also associated with less cardiac inflammation [44]. These anti-inflammatory effects of sildenafil, corroborated by Rizzo et al. [45], consequently indirectly further support the role of cardiac inflammation in cardiac remodeling and LV performance. Furthermore, these findings confirm that besides cardiomyocytes, also fibroblasts [46] and endothelial cells [45] are target cells of sildenafil. Despite abovementioned promising experimental findings [44], sildenafil failed to raise cGMP levels and to ameliorate LV diastolic function in HFpEF patients [47].

#### Endothelial-to-Mesenchymal Transition

Thirdly, NO deprivation [20, 48] leads to endothelial-to-mesenchymal transition (EndMT), a process whereby endothelial cells convert to a mesenchymal cell type, which can give rise to fibroblasts. EndMT is induced by inflammatory factors (TGF-ß [49] and TNF-α [50]), oxidized LDL [51], and age [50]. In contrast, we could demonstrate that HDL decrease EndMT. Zeisberg et al. (2007) [49] provided first evidence that EndMT contributes to cardiac fibrosis during chronic pressure overload. Recently, endothelial-specific expression of endothelin-1 [52] and of NOX 2 [31] has been shown to induce EndMT in experimental models of diabetes mellitus and Ang II-induced cardiac hypertrophy and fibrosis, respectively. Intriguingly, Ang II-induced endothelial NOX2 activation was associated with isolated diastolic dysfunction in the absence of systolic dysfunction.

In summary, these findings suggest that endothelial dysfunction/activation and downstream pathways including not only inflammation but also far less appreciated EndMT, are important mechanisms contributing to cardiac fibrosis and the development of HFpEF [31]. Cardiac fibroblasts in turn can further trigger cardiac inflammation [53] in a multimodal manner which is reviewed in detail elsewhere [32••]. This complex interaction among endothelial cells, inflammatory cells, and cardiac fibroblasts might explain why unidirected strategies [54] counteracting inflammation or fibrosis have failed so far to block the fibrotic process and indicates the need for new strategies with more broaden immunomodulatory effects [32••]. Furthermore, it stresses the need of diagnosing and treating HFpEF at an early stage of its pathogenesis, where the vicious circle of inflammation and fibrosis, leading to chronic inflammation, might still be abrogated.

### Impact of Endothelial Dysfunction on Cardiomyocytes

The endocardial endothelial cells and the endothelial cells of intramyocardial capillaries regulate the contractile state of cardiomyocytes via autocrine and paracrine signaling involving NO and endothelin-1. Furthermore, co-culture of cardiac endothelial cells with cardiomyocytes protects those cardiomyocytes from hydrogen peroxide-induced apoptosis through neuregulin-erbB4 signaling [55]. This indicates that cardiac endothelial cells exert direct anti-apoptotic effects in addition to their role in oxygen supply, which is needed for cardiomyocyte survival. Cardiac endothelial cells also guide cardiomyocyte organization and promote physiological coupling of cardiomyocytes and their synchronized contraction via influencing the expression of the principal gap junction protein connexin 43 [56]. On the other hand, upon inflammation [57] and mechanical load [58, 59], endothelial cells may contribute to cardiomyocyte hypertrophy via the release of endothelin-1. Cardiomyocytes in turn can affect the coronary vasculature via multiple paracrine signals including endothelin-1 and fibroblast growth factor 2 [60, 61]. In addition, cardiomyocytes affect long-term growth and development of coronary arterial, venous, and lymphatic trees in which the paracrine release of VEGF-A by cardiomyocytes is of particular importance [60, 62]. This cardiomyocyte-vascular crosstalk plays a critical role in the vascular adaptation, which takes place during myocardial hypertrophy. An imbalance between vasculature and cardiomyocyte growth may lead to progressive cardiac dysfunction and heart failure [60]. In brief, the above findings indicate the delicate balance between endothelial cells and cardiomyocytes and imply that changes due to endothelial dysfunction or deterioration may contribute to cardiomyocyte hypertrophy and HFpEF.

Particularly, the contribution of cardiomyocyte stiffness in diastolic LV stiffness and HFpEF is established. Besides intracellular calcium dysregulation, involving dysfunction of the sarcoplasmic reticular adenosine triphosphate (ATP)-driven pump (SERCA), phospholamban (PLB), and/or ryanodine receptor (RYR) 2, cardiomyocyte stiffness is mainly regulated by the giant sarcomeric protein titin [63]. Titin spans the sarcomere from the Z disk to the M line and functions as a molecular spring supporting early diastolic recoil and late diastolic distensibility of cardiomyocytes [64]. Evidence that solely titin stiffness is sufficient to induce diastolic dysfunction and HFpEF, independent of extensive collagen deposition, follows from recent experimental studies. Chung et al. generated mice with a deletion of nine immunoglobulin-like domains from the proximal tandem immunoglobulin segment of the titin spring region, which resulted in overall titin stiffness [6•]. These knockout mice developed HFpEF despite unaltered myocardial collagen content. In addition, Hamdani et al. [65] recently demonstrated the importance of titin stiffness in the induction of diastolic dysfunction independently of cardiac fibrosis in a rat model of the metabolic syndrome. Besides shortage of titin length by experimental deletion of immunoglobulin-like domains [6•] or by oxidative stress-induced formation of disulfide bridges within the titin molecule [66], titin stiffness is also attributed to isoform shifts or posttranscriptional modifications like phosphorylation or oxidation [64]. Protein kinase Cα phosphorylates titin at its proline, glutamate, valine, and lysine (PEVK) titin region and raises the stiffness of cardiomyoctes from normal myocardium [67], but not from cardiomyocytes isolated from an animal model of HFpEF [68]. On the other hand, the protein kinase extracellular signal-regulated kinase 2 [69] and Ca^2+^/calmodulin-dependent protein kinase II [70] phosphorylate titin and lower cardiomyocyte stiffness, though their pathophysiological relevance for HFpEF needs to be further clarified. In HFpEF patients, titin at the stiff N2B isoform is hypophosphorylated [71]. Protein kinases A [72] and G [73, 74••] phosphorylate titin at its N2B segment, and lower cardiomyocyte stiffness, which implies that ß adrenergic stimulation, NO, and natriuretic peptides may decrease cardiomyocyte stiffness. With respect to Ca^2+^/calmodulin-dependent protein kinase II and its involvement in NO synthesis as a result of Ca^2+^-dependent activation of eNOS [75], it is tempting to speculate that Ca^2+^/calmodulin-dependent protein kinase II may further contribute to the relaxation of titin via triggering NO release. Due to the potential risk of arrhythmic death, the induction of protein kinase A via ß adrenergic stimulation is excluded as therapeutical option. Blocking the breakdown of the downstream NO target cGMP via sildenafil has not been successful in HFpEF patients despite promising experimental studies (see infra). In contrast, favorable effects in improving diastolic dysfunction in patients with HFpEF have recently been found by the use of the neprilysin inhibitor (LCZ696), which inhibits the breakdown of natriuretic peptides. Neprilysin activity is induced in obese patients [76]. The finding that IL-1ß induces neprilysin activity [77] may explain the increased neprilysin and B-type natriuretic peptide (BNP) paradox in obese HFpEF patients, which is associated with increased inflammation [78].

In summary, the importance of NO bioavailability and oxidative stress in titin relaxation (of the N2B segment) [74••] as well as the role of inflammation in the induction of neprilysin activity [77] and subsequent impaired natriuretic signaling further corroborates the impact of endothelial dysfunction on cardiomyocyte stiffness.

### Impact of Endothelial Dysfunction on Cardiac Neurohumoral Regulation

As it has already been broadly outlined before, HFpEF comorbidities provoke a systemic inflammatory state, which severely affects the coronary microvascular endothelium [36, 79]. The sum outcome of microvascular deterioration is neuronal dysfunction and death as well as the reduction of neurovascular perfusion, functional impairment, and cellular apoptosis [80]. Inflammatory and hyperglycemia-induced oxidative stress in vascular endothelial cells correlates with cardiac autonomic nerve dysfunction [81]. eNOS uncoupling leading to nitrosative stress and formation of peroxynitrite induces damage of neuronal axons, which are particularly prone to oxidative and nitrosative stress due to their high mitochondrial content [82]. This leads to a prominent abnormality in the levels of cardioactive neuropeptides including neuropeptide Y, substance P [83], calcitonin-generated peptide, natriuretic peptides (ANP, BNP, and C-type natriuretic peptide) [84] as well as the respective neuropeptide effectors [85], contributing to impaired endothelium-dependent vasodilation in HFpEF [86]. This limits the delivery and extraction of tissue oxygen, which subsequently results in nerve hypoxia and impaired nerve function [80]. The damage of the nerve fibers innervating the heart and blood vessels, common for cardiovascular autonomic neuropathy, results in abnormalities in heart rate control and vascular dynamics. Besides their role as vasodilatory proteins, the natriuretic peptides ANP and BNP exert anti-fibrotic [87] and anti-hypertrophic effects [88] and influence titin stiffness. Under metabolic conditions, BNP degradation seems to be paradoxically increased [89], contributing to cardiac fibrosis and cardiomyocyte stiffness.

Cardiovascular autonomic neuropathy in diabetes mellitus is associated with LV diastolic and systolic dysfunction, LV hypertrophy, and higher LV mass index [90]. Findings from studies of the autonomic nervous system indicate that it can modulate inflammatory reactions [91]. These findings suggest that dysregulation of the cardiac neurohumoral system not only directly modulates vascular dynamics and heart function but also activates cardiac inflammation and subsequent remodeling.

## Conclusions and Perspectives

HFpEF is a complex disorder caused by multifactorial stresses secondary to comorbidities. This review highlights the importance of endothelial dysfunction or deterioration at the early onset of the pathogenesis of HFpEF and stresses the subsequent complexity of cardiac remodeling due to bidirectional interactions between endothelial cells, cardiomyocytes, fibroblasts, and cardiac neurohumoral activation. It further strengthens the need for an early diagnosis of endothelial dysfunction [92••] enabling treatment before the development of HFpEF-specific cardiac remodeling. So far, only the prevention of HFpEF through treatment of risk factors has been shown to be effective [93]. It will be the challenge of finding new multidirectional strategies to abrogate endothelial dysfunction and subsequent cardiac remodeling. Given the importance of inflammation in the induction of endothelial dysfunction and cardiac remodeling via the cardiosplenic axis [33, 34], intravenous application of mesenchymal stromal cells, and the recently identified cardiac-derived adherent proliferating cells (CardAPs) [94] having immunomodulatory [34, 95–97], endothelium-protective [98], and anti-fibrotic [34, 99, 100] features might be attractive tools to counteract the inflammatory and cardiac remodeling process.

## Abbreviations

Ang II: Angiotensin II
ANP: Atrial natriuretic peptide
BNP: B-type natriuretic peptide
cAMP: Cyclic adenosine monophosphate
cGMP: Cyclic guanosine monophosphate
EndMT: Endothelial-to-mesenchymal transition
HFpEF: Heart failure with preserved ejection fraction
LV: Left ventricular
MMP: Matrix metalloproteinase
MSC: Mesenchymal stromal cell
NADPH: Nicotinamide adenine dinucleotide phosphate
NOX: NADPH oxidase
PDE 2: Phosphodiesterase 2
TNF: Tumor necrosis factor
TGF: Transforming growth factor
VCAM: Vascular cell adhesion molecule
